# Quality Assessment of Medical Institutions’ Websites Regarding Prescription Drug Misuse of Glucagon-Like Peptide-1 Receptor Agonists by Off-Label Use for Weight Loss: Website Evaluation Study

**DOI:** 10.2196/68792

**Published:** 2025-01-01

**Authors:** Rie Oyama, Tsuyoshi Okuhara, Emi Furukawa, Hiroko Okada, Takahiro Kiuchi

**Affiliations:** 1 Department of Health Communication School of Public Health Graduate School of Medicine, The University of Tokyo Tokyo Japan; 2 University Hospital Medical Information Network The University of Tokyo Hospital Tokyo Japan

**Keywords:** prescription drug misuse, GLP-1 receptor agonists, off-label use, weight loss, information quality, DISCERN, web-based information, information provision, misinformation, advertising guidelines, exaggerated advertisements

## Abstract

**Background:**

Misuse of glucagon-like peptide-1 receptor agonists (GLP-1RAs) has emerged globally as individuals increasingly use these drugs for weight loss because of unrealistic and attractive body images advertised and shared on the internet.

**Objective:**

This study assesses the quality of information and compliance with Japan’s medical advertising guidelines on the websites of medical institutions that prescribe GLP-1RAs off-label for weight loss.

**Methods:**

Websites were identified by searching Google and Yahoo! by using keywords related to GLP-1RAs and weight loss in August 2024. The quality of information on these websites was assessed using the DISCERN instrument. To comply with Japan’s medical advertising guidelines, we evaluated whether the 5 mandatory items for advertisements of self-paid medical treatments involving the off-label use of drugs were stated and whether there were any exaggerated claims. The content of the exaggerated advertisements was categorized into themes.

**Results:**

Of the 87 websites included, only 1 website stated all 5 mandatory items. Websites listing “ineligible for the relief system for sufferers from adverse drug reactions” had the lowest percentage at 9% (8/87), while 83% (72/87) of the websites listed exaggerated advertisements. Approximately 69% (60/87) of the websites suggested that no exercise or dietary therapy was required, 24% (21/87) suggested that using GLP-1RAs is a natural and healthy method, and 31% (27/87) of the websites provided the author’s personal opinions on the risks of using GLP-1RAs. The mean total DISCERN score for all 87 websites was 32.6 (SD 5.5), indicating low quality. Only 1 website achieved a good rating, and 9 websites were rated as fair. The majority of the websites were rated as poor (72 websites) or very poor (5 websites).

**Conclusions:**

We found that the quality of information provided by the websites of medical institutions prescribing GLP-1RAs off-label for weight loss was very low and that many websites violated Japan’s medical advertising guidelines. The prevalence of exaggerated advertisements, which may lead consumers to believe that they can lose weight without dietary or exercise therapy, suggests the risk of GLP-1RA misuse among consumers. Public institutions and health care providers should monitor and regulate advertisements that violate guidelines and provide accurate information regarding GLP-1RAs, obesity, and weight loss.

## Introduction

Prescription drug misuse is an emerging global public health issue [1-3]. According to a previous study [4], misuse is defined as the therapeutic use of legal drugs in a manner other than as prescribed, and abuse is defined as the use of illegal drugs for nontherapeutic purposes such as psychological pleasure and weight loss. The misuse and abuse of drugs can lead to health problems. In the United States and European countries, misuse of opioid analgesics, benzodiazepines, over-the-counter cough suppressants containing dextromethorphan and codeine, and psychostimulants have been observed [1-3,5]. Further, in Japan, misuse of over-the-counter cough suppressants, pain relievers, antianxiety medications, and sedative drugs has been observed [6,7]. However, misuse of glucagon-like peptide-1 receptor agonists (GLP-1RAs) has become a problem in recent years [8-10].

GLP-1RAs are the most effective drugs for obesity treatment [11]. Semaglutide (Wegovy), a type of GLP-1RA, was approved by the US Food and Drug Administration (FDA) as an obesity treatment drug in 2021 [12]. Since then, its market share has rapidly expanded [13]. Conversely, multiple news media outlets have reported concerns about the misuse of GLP-1RAs [14,15]. Due to unrealistic and attractive body images that are advertised by celebrities on social media, people without obesity allegedly obtain and use GLP-1RAs illicitly for weight loss purposes [16]. Adverse events of GLP-1RA misuse have also been reported [8,9]. A previous study showed that the use of semaglutide in patients with overweight/obesity may be associated with the incidence of nonarteritic anterior ischemic optic neuropathy, a severe ophthalmologic condition that can lead to blindness [17]. Another previous study showed that GLP-1RA users for weight management had an increased risk of serious gastrointestinal disorders compared to bupropion-naltrexone users, with adjusted hazard ratios for pancreatitis, bowel obstruction, and gastroparesis reported as 9.09, 4.22, and 3.67, respectively [18]. However, the use of GLP-1RAs for weight management is a relatively new therapeutic approach, and the long-term side effects associated with their use remain unknown. Additionally, the effectiveness and potential adverse effects of using GLP-1RAs for further weight loss in individuals without obesity also remain unknown. This increased demand has caused a global shortage of GLP-1RAs [16]. The European Medicines Agency has announced recommendations by manufacturers, health care professionals, and citizens for the proper use of GLP-1RAs [19].

In Japan, Wegovy was approved for the treatment of obesity on March 27, 2023. According to the Optimal Use Promotion Guideline by the Japan’s Ministry of Health, Labor, and Welfare, patients eligible for obesity treatment are “those with BMI ≥35 kg/m² or those with BMI ≥27 kg/m² who also have 2 or more health conditions related to obesity and who do not achieve sufficient results from diet and exercise therapy” [20]. The guidelines also strictly prohibit the use of GLP-1RAs for weight loss purposes without obesity as well as institutional criteria for prescribing GLP-1RAs under insurance-covered medical care [20,21]. Japan’s Ministry of Health, Labor, and Welfare released a “Request for cooperation due to tight inventory of GLP-1 receptor agonist” [22], and the Japan Diabetes Society [23] and Japan Society for the Study of Obesity [24] have made recommendations regarding the off-label use of GLP-1RAs.

However, several private clinics in Japan prescribe GLP-1RAs for off-label weight loss. Japan’s National Consumer Affairs Center and Consumer Affairs Agency warns citizens against the use of GLP-1RAs for weight loss purposes because of the possibility of medical institutions prescribing GLP-1RAs through advertisements that are prohibited by guidelines, inadequate physician consultations and explanations, and inadequate systems to manage adverse events [25,26]. Websites of medical institutions in Japan are regulated by the Medical Care Act [27] and the Ministry of Health, Labor and Welfare’s “Regulations on Advertisements of Hospitals, etc under the Medical Care Act” (Japan’s medical advertising guidelines) [28], which prohibit false or exaggerated advertisements. The reason for this is “because false advertisements may cause patients to lose the opportunity to receive appropriate medical consultation or receive inappropriate medical treatments by giving them information that is significantly different from the facts, etc”[28].

In previous studies on health information quality, these issues were assessed as part of the reliability of health information. The DISCERN instrument developed by Charnock et al [29] has been used in many studies to assess the publication reliability and the quality of the written health information (Multimedia Appendix 1). The validity and reliability of the DISCERN instrument were ensured, and in addition to the original English language version, Chinese [30], Spanish [31], Brazilian Portuguese [32], and Japanese [33] versions have been developed [29]. Previous studies using the DISCERN instrument have examined the quality of information on websites regarding posttraumatic stress disorder [34], breast cancer [35], nasopharyngeal carcinoma [36], and cosmetic injectability [37]. These studies showed that the quality of individual websites varied considerably: the quality of information was higher for academic and government institutions that operate websites and lower for commercial institutions [34-37]. A systematic review showed that in research using the DISCERN instrument, none found the mean DISCERN score of all websites to be excellent, and the information quality of the majority of websites ranged from fair to very good [38].

In recent years, most people have searched the internet for health information [39]. The internet usage rate for individuals in Japan in 2022 was 84.9% and exceeded 90% when limited to the 10-50 years age range [40]. For cosmetic treatments, 95% of the patients search for information on the internet before entering a doctor’s office [41]. Hence, if medical institutions’ websites on GLP-1RAs contain misleading, exaggerated, or unreliable information, these websites should be immediately assessed and improved. However, to our knowledge, no study has investigated the quality of information that people without obesity obtain from websites when they consider obtaining GLP-1RAs for weight loss. This study aims to assess compliance with medical advertising guidelines and the quality of information on the websites of medical institutions that prescribe GLP-1RAs off-label for weight loss in Japan.

## Methods

### Data Collection

#### Search Terms

We used a Japanese-language search string input into Google Japan [42] and Yahoo Japan [43], which has the largest search engine market shares in Japan, in August 2024 [44]. The GLP-1RAs approved by the FDA for obesity treatment are liraglutide, semaglutide, and tirzepatide that are sold in Japan under the product names Rybelsus, Ozempic, Victoza, Saxenda, and Mounjaro. The search keywords comprised 6 in total (Rybelsus diet, Ozempic diet, Victoza diet, Saxenda diet, Mounjaro diet, and GLP-1 diet), which included each product name combined with diet (used specifically to mean weight loss in Japan), as well as the comprehensive term GLP-1 diet. Wegovy was excluded from the search terms because it was the newest drug and was not available in the general market in Japan. Saxenda is approved in Japan for the treatment of diabetes under the name Victoza, but private clinics that use the drug off-label advertise it under the name Saxenda; so, it was included in the search terms.

All words were entered in the search window, one keyword at a time, and 20 websites were reviewed per search engine for each term. This is because a previous study showed that 97.2% of the users only view the first 1-10 websites of search results, and ˂2% click on links on the second page of the search results and beyond [45]. Google Chrome’s incognito browser that would have all history deleted was used, and personal accounts were not used.

#### Inclusion and Exclusion Criteria

The inclusion criteria were as follows: websites written in Japanese; websites created by medical institutions (with medical department labels); websites clearly stating that GLP-1RAs were being sold; and the medical institutions operating the websites were not accredited as facilities capable of treating obesity using GLP-1RAs by the Japanese Circulation Society, Japan Diabetes Society, or Japan Endocrine Society. The exclusion criteria were as follows: duplicate websites, government or academic statements, PDF documents, news reports, affiliate advertisements, and websites that were unclear whether they prescribe GLP-1RAs. The first author (RO) reviewed all the websites in the search results and extracted eligible websites.

### Data Extraction

RO recorded the names and URLs of all the websites, names of the medical institutions operating the websites, the medical departments they represented, and the types of GLP-1RAs they sold. If not listed, it was recorded as unknown. Medical institutions can represent multiple medical departments in Japan. Therefore, if several departments were listed, all were recorded. All data were recorded in a Microsoft Excel 2021 spreadsheet by RO.

### Data Assessment

First, all identified websites were assessed by an internal medicine physician (RO) for compliance with the medical advertising guidelines in Japan and quality by using the DISCERN instrument. Second, the third author (EF, an internal physician) was trained through a 1-hour meeting and a 1-hour pilot assessment, and then EF independently assessed approximately 20% (20/87) of the total websites. These 20 websites were randomly selected using Microsoft Excel to generate random numbers. Finally, they assessed the agreement between the coders of the 20 websites. The results were recorded using a Microsoft Excel 2021 spreadsheet. More precise information is provided below.

### Assessment of Compliance With Medical Advertising Guidelines

In the case of noninsured medical treatments involving off-label use of drugs, it is prohibited to advertise on websites without explicitly stating that “the drugs are not approved, the acquisition route, information on whether there are other domestically approved drugs with the same ingredients and performance, information on the safety of the products in other countries, and they are ineligible for the relief system for sufferers from adverse drug reactions,” in Japan’s medical advertising guidelines [28]. All the websites were assessed based on whether these 5 items were stated.

Japan’s medical advertising guidelines also prohibit exaggerated advertisements [28]. The guidelines [28] define exaggerated advertisements as advertisements that are not necessarily false but that misleads citizens by misrepresenting facts such as about the size of the facility, staffing, or the contents of the medical care provided. Moreover, “misleading citizens” is defined as “it is sufficient to say, as a matter of common sense, that there is a difference between the ‘impression’ or ‘expectation’ that citizens perceive from the advertisements and the actual contents, and it is not necessary to prove that the advertisements mislead or to show that the advertisements have actually misled citizens” [27]. Therefore, since taking GLP-1RAs is considered an auxiliary therapy for exercise and dietary therapy in the treatment of obesity, statements such as “you can lose weight with GLP-1RAs alone,” “you can lose weight even if you cannot exercise,” or “you do not need dietary therapy” are considered exaggerated advertisements. The coders carefully read the guidelines and assessed each statement by using a binary (0/1) scale to indicate the presence or absence of exaggerated advertisements. RO then analyzed the contents of the exaggerated advertisements and categorized them into themes according to the specific examples in the guidelines [28].

### Quality Assessment Using the DISCERN Instrument

The DISCERN instrument in the Japanese version [33] was used for the quality assessment of information. The DISCERN instrument consists of 16 items, each of which is assessed on a 5-point Likert scale (1=not at all, 5=completely). Section 1 (questions 1-8) addresses website reliability, section 2 (questions 9-15) addresses the quality of information about treatment options, and section 3 (question 16) presents the overall assessment. The total score ranged from 16 to 80, with higher scores indicating higher quality information. Several previous studies ranked the quality of websites based on their total score as follows: excellent=80-63, good=62-51, fair=50-39, poor=38-27, very poor=26-16 [35,46].

### Statistical Analysis

Data are presented as means for continuous variables and counts with frequency percentages for categorical variables. Cohen κ statistic was used to measure the intercoder reliability for compliance with medical advertising guidelines and DISCERN scores between the first and second coders. Linear regression models were used to investigate the association between the specialties of the clinic operating the website and the total DISCERN score. *P* values were 2-sided, and *P*<.05 was considered statistically significant. All analyses were conducted using the R version 4.3.1 (June 16, 2023; R Foundation for Statistical Computing).

### Ethical Considerations

This study did not involve human or animal participants, and all data were collected from publicly available web-based sources. This study was granted an exemption from ethics approval by the ethics review committee of the Graduate School of Medicine, University of Tokyo.

## Results

### Characteristics of the Included Websites

**Figure 1:**
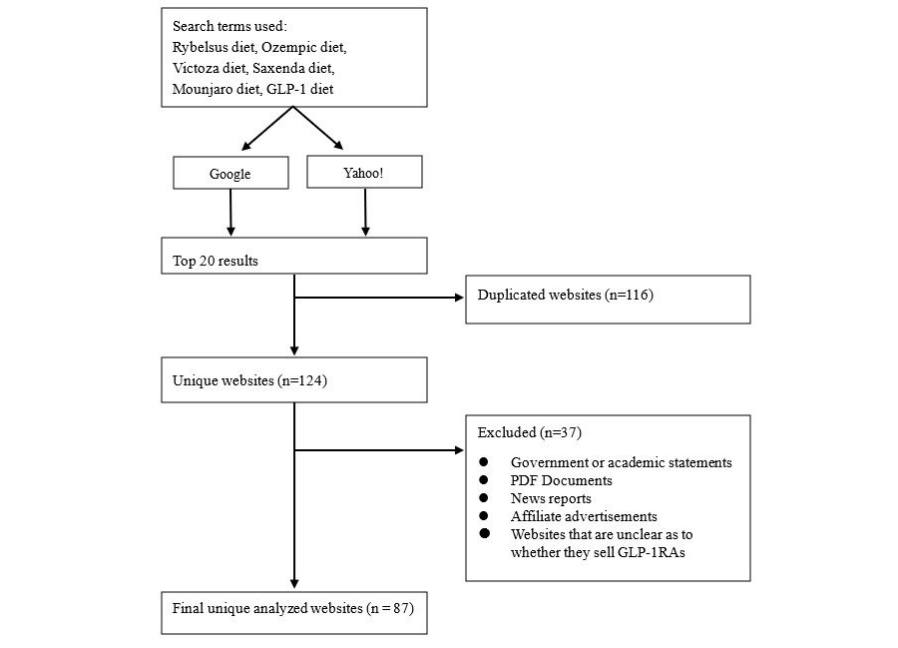
Search process flowchart. GLP-1: glucagon-like peptide-1; GLP-1RA: glucagon-like peptide-1 receptor agonist.

In total, 240 websites were identified. After the removal of intrasearch term duplicates and those that met the exclusion criteria, 87 websites were used for the analysis (Figure 1). A total of 67 clinics operated the 87 websites. This was because 8 clinics had multiple different GLP-1RA diet-related articles. Table 1 shows that the most common departments, as far as could be determined from the websites, were dermatology (22/120, 18.3%), cosmetic dermatology (21/120, 17.5%), and internal medicine (19/120, 15.8%); 17 clinics were unaware of their departments. The percentages of advertisements with Ozempic, Rybelsus, Mounjaro, Victoza, and Saxenda were 18.5% (30/162), 29.1% (47/162), 12.3% (20/162),14.2% (23/162), and 25.9% (42/162), respectively. Approximately 18% (16/87) of the websites mentioned selling sodium-glucose cotransporter 2 inhibitor along with GLP-1RAs, and 12% (10/87) mentioned selling metformin.

**Table 1:** Characteristics of the websites regarding prescription drug misuse of glucagon-like peptide-1 receptor agonists by off-label use for weight loss.

		Values, n (%)
**Specialty^a^ (n=120^b^)**		
	Dermatology	22 (18.3)
	Cosmetic dermatology	21 (17.5)
	Internal medicine	19 (15.8)
	Cosmetic surgery	6 (5)
	Urology	6 (5)
	Allergology	5 (4.2)
	Other	24 (20)
	Unknown	17 (14.2)
**Types of advertised drugs^c^ (n=162^d^)**		
	Ozempic	30 (18.5)
	Rybelsus	47 (29)
	Mounjaro	20 (12.3)
	Victoza	23 (14.2)
	Saxenda	42 (25.9)

^a^Medical departments represented by medical institutions.

^b^Total number of mentions of specialty.

^c^Types of glucagon-like peptide-1 receptor agonists that medical institutions advertised for sale.

^d^Total number of mentions of drugs.

### Compliance With Medical Advertising Guidelines

The intercoder reliability was acceptable (average Cohen κ=0.667). Table 2 shows the compliance with medical advertising guidelines. Only one of the 87 websites stated the 5 required items. The items with the highest percentage of listing were “acquisition route” and “information on the safety of the drugs in other countries,” both at 79% (69/87). The item with the lowest percentage of listing was “ineligible for the relief system for sufferers from adverse drug reactions” at 9% (8/87).

**Table 2:** Compliance with advertising guidelines of the websites regarding prescription drug misuse of glucagon-like peptide-1 receptor agonists by off-label use for weight loss (N=87).

		Values, n (%)
**Content that should be included^a^**		
	The drug is unapproved	43 (49)
	Acquisition route	69 (79)
	Information on whether there are other domestically approved drugs with the same ingredients and performance	24 (28)
	Information on the safety of the drug in other countries	69 (79)
	Ineligible for relief system for sufferers from adverse drug reactions	8 (9)
Exaggerated advertisements^b^		72 (83)

^a^Explicit statements required under Japan’s medical advertising guidelines for noninsured medical treatments involving off-label drug use.

^b^Advertisements containing expressions that could mislead citizens, as determined by coders under Japan’s medical advertising guidelines.

Table 2 shows that 83% (72/87) of the websites listed exaggerated advertisements. Table 3 shows the content of the exaggerated advertisements: of the 87 websites, 69% (60/87) conveyed messages indicating no exercise and dietary therapy were required, 24% (21/87) conveyed messages indicating that using GLP-1RAs is a natural and healthy method, and 31% (27/87) provided the website author’s subjective expression of the risks of using GLP-1RAs.

**Table 3:** Content of exaggerated advertising regarding prescription drug misuse of glucagon-like peptide-1 receptor agonists by off-label use for weight loss (N=87)^a^.

Themes	Illustrative quotes	Values, n (%)
Induction by nonscientific information (no diet or exercise therapy required)	…*Exercise is also unnecessary. Exercise is less effective in losing weight.* …*Medical dieting is a treatment that suppresses appetite and promotes weight loss by taking a medicine called GLP-1 receptor agonist, which is used in the treatment of diabetes, without diet restrictions or hard exercise regimen.*	60 (69)
Induction by nonscientific information (natural and healthy methods)	…*By managing portion sizes, you can reduce calorie intake and engage in a natural and controlled dietary regimen.* …*To achieve weight loss in a healthy manner.* …*It also has fat-burning and pancreas-protecting effects, making it a healthy way to lose weight.*	21 (24)
Subjective expressions of risks	…*Since the delayed gastric emptying effect is not long-lasting, there is little need to consider it dangerous.* …*I, as the director, have also used it and had no side effects other than slight heartburn, which may have caused mild anorexia and slight weight loss.*	27 (31)
Statements that you can always get the drug only through web-based medical consultation	…*Everything from making an appointment and consultation to prescribing a drug is done online, so there is no need to go out.* …*You can see us from anywhere in the country via our web-based clinic. [...] There is no consultation fee; GLP-1 injections will be mailed to you.*	20 (23)

^a^Advertisements containing expressions that could mislead citizens, as determined by coders under Japan’s medical advertising guidelines.

### Quality Assessment Using DISCERN Instrument

**Figure 2:**
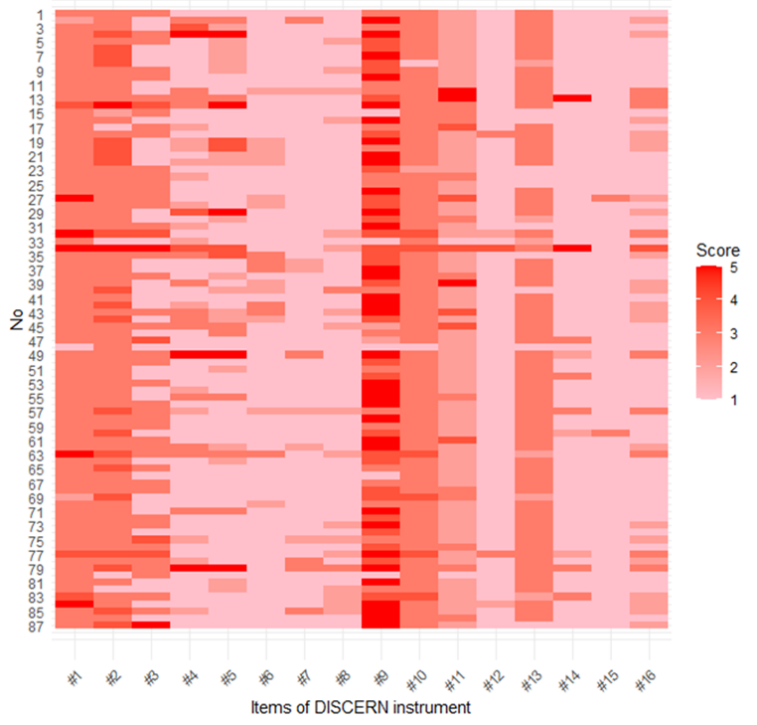
DISCERN scores for each item: #1 to #16 represent the criteria numbers of the DISCERN instrument. No: identification numbers for individual websites for analysis.

Intercoder reliability was acceptable (average Cohen κ=0.663). Figure 2 shows the DISCERN scores for all 87 websites across each item. Table 4 presents the mean score for each of the 16 DISCERN instrument criteria and the total DISCERN score. The mean total DISCERN score for all 87 websites was 32.6 (SD 5.5). Only 1 website scored more than 51 points, which is good; 9 websites scored between 39 and 50 points, which is fair; 72 websites were poor; and 5 websites were very poor. The items that scored a mean of at least 3 points were criterion 9 (does it describe how each treatment works?) with a mean score of 3.96 (SD 1.10), criterion 2 (does it achieve its aims?) with a mean score of 3.18 (SD 0.70), criterion 1 (are the aims clear?) with a mean score of 3.10 (SD 0.57), and criterion 10 (does it describe the benefits of each treatment?) with a mean score of 3.01 (SD 0.42). The items that scored a mean of less than 2 points were criterion 15 (does it provide support for shared decision-making?), criterion 12 (does it describe what would happen if no treatment was used?), criterion 7 (does it provide details of additional sources of support and information?), criterion 8 (does it refer to the areas of uncertainty?), criterion 14 (is it clear that there may be more than one possible treatment choice?), criterion 6 (is this balanced or unbiased?), criterion 4 (is it clear what sources of information were used to compile the publication?), and criterion 5 (is it clear when the information used or reported in the publication was produced?). The mean score for criteria 16, which assessed the overall quality, was 1.49 (SD 0.73). No statistically significant association was found between the specialties and the total DISCERN score (all *P*>.05).

**Table 4:** Mean score for each of the 16 DISCERN instrument criteria^a^.

Criterion number	Criteria	Mean score (SD)
1	Provides clear aims	3.10 (0.57)
2	Achieves its aims	3.18 (0.71)
3	Provides relevant information	2.21 (1.15)
4	Provides sources of information	1.70 (1.07)
5	Provides information production date	1.73 (1.16)
6	Balanced and unbiased	1.28 (0.58)
7	Provides additional sources of information	1.18 (0.52)
8	Refers to areas of uncertainty	1.24 (0.48)
9	Describes how each treatment works	3.97 (1.10)
10	Describes benefits of each treatment	3.01 (0.42)
11	Describes risks of each treatment	2.36 (0.79)
12	Describes what would happen if no treatment is used	1.10 (0.46)
13	Describes how treatment affects quality of life	2.70 (0.67)
14	Makes clear that there may be more than one possible treatment choice	1.24 (0.76)
15	Supports shared decision-making	1.05 (0.30)
16	Overall quality	1.49 (0.73)

^a^Each DISCERN item is assessed on a 5-point Likert scale (1=not at all, 5=completely). Total score = 32.55 (SD 5.53).

## Discussion

### Principal Findings

This study assesses compliance with Japanese medical advertising guidelines, exaggerated advertisement classification, and DISCERN scores of Japanese websites of medical institutions that prescribe GLP-1RAs off-label for weight loss, which Japanese consumers may refer to when considering weight loss with GLP-1RAs.

Many websites potentially violated Japan’s medical advertising guidelines, and very few websites listed all the items required for advertising GLP-1RAs off-label use. In particular, only 9% (8/87) of the websites stated that using GLP-1RAs off-label was not covered by the Adverse Reactions Relief System for Drugs. In recent years, problems related to cosmetic therapy for weight loss have increased in Japan [47], so it is problematic that consumers have limited exposure to information indicating that the off-label use of GLP-1RAs is not covered by the Adverse Reactions Relief System for Drugs. Furthermore, concerns have been raised that GLP-1RAs may be obtained without a prescription and are at risk of misuse and abuse in inappropriate quantities [8]. However, research on who is actually misusing and what kinds of adverse effects occur because of misuse remains scarce. Generally, it has been reported that distinguishing between therapeutic errors, unintentional misuse, and intentional abuse of drugs is challenging, and the content of the reports tends to vary [5]. As mentioned above, in Japan, because it is not covered by the Adverse Reactions Relief System for Drugs, it may be difficult for consumers who have experienced adverse events due to the misuse of GLP-1RAs to claim compensation.

Approximately 83% (72/87) of the websites included exaggerated advertisements, and much of their content may mislead consumers into assuming that one can lose weight by using GLP-1RAs without exercise or dietary therapy. However, since participants in clinical trials evaluating the efficacy of obesity treatment drugs received medication therapy as an auxiliary to lifestyle interventions, the message that the use of GLP-1RAs for weight loss does not require exercise or dietary therapy is unscientific [48]. Additionally, according to another previous study, such messages may evoke a “compensatory health belief” in consumers that “if an alternative behavior is adopted, it is not necessary to engage in healthy behaviors or refrain from unhealthy ones.” [49,50]

In this study, the mean of the total DISCERN scores of the analyzed websites was 32.56, which qualified as poor. Individually, most of the websites qualified as poor, only 1 website qualified as good, 9 websites qualified as fair, 72 websites qualified as poor, and 5 websites qualified as very poor. The mean score of criterion 16, the overall quality rating, was also low (score=1.49). These results were extremely low compared to those of previous studies that used the DISCERN instrument to assess the quality of health information [34-36,38]. For each question, criterion 15 (shared decision-making), criterion 12 (if no treatment is used), criterion 7 (additional information sources), criterion 8 (uncertainty), criterion 14 (possible treatment choice), and criterion 6 (balanced and unbiased) scored low, and criterion 9 (how each treatment works), criterion 2 (achieves its aims), and criterion 1 (provides clear aim) scored high. This trend was consistent with that of previous studies [34-36]. Unlike previous studies that compared DISCERN scores by the type of website operator (academic, government, or commercial agencies), only private clinic websites that prescribe GLP-1RAs for off-label use were assessed in this study. Therefore, the DISCERN score was significantly lower in this study, possibly because the purpose of the websites was to sell GLP-1RAs and not to provide accurate information about obesity or obesity treatment to the public. In other words, these websites were advertisements designed to appeal to consumers about the effectiveness of GLP-1RAs for weight loss and not to draw their attention to other treatment options or shared decision-making with other health care providers or family members. Nevertheless, a previous study on consumer judgments of the reliability of web-based health information showed that website owners’ authority had a positive impact on reliability and credibility [51]. Accordingly, if medical institutions representing specialties such as internal medicine were the owners of these websites, many consumers might trust the content of the websites. In summary, the problems with the information on medical institution websites identified by this study are not only unscientific and in violation of medical advertising guidelines but are also problematic from the perspective of public health and health promotion.

In this study, we observed that there were few public institution websites providing information on obesity treatment for consumers. To the best of our knowledge, no study has comprehensively compared the overall quality of web-based medical information between Japan and other countries. However, a previous study comparing palliative care websites in the United States and Japan reported that Japanese websites were of lower quality than the American ones [52]. A study focusing on the quality of web-based medical information within Japan has generally reported low quality [53]. This issue may be influenced by differences in the medical advertising regulations between Japan and other countries. Future studies should aim to provide a more comprehensive and international assessment of the quality of medical information.

### Practical Implications

The results of this study have several implications. Regarding future studies, researchers should assess the reliability of media content, including medical institutions’ websites that prescribe drugs for off-label use to contribute to better control of prescription drug misuse in the future. Many studies have investigated the consumer risk factors and preventive interventions for drug misuse. For example, a study investigating the misuse of over-the-counter drugs showed that health literacy, low education level, and misunderstanding of over-the-counter medicine instructions were correlated with the incidence of misuse [5]. Another study showed that the misuse of prescription stimulants for weight loss was positively associated with being female, lacking insurance, and having a history of lifetime misuse but not past-year misuse of prescription sedatives or tranquilizers [54]. Limited health literacy has been reported to be correlated with overweight/obesity in the first place [55], greater difficulty in the medication compliance, and higher mortality [56]. Thus, efforts to enhance consumer health literacy may be equally important as well as the content reliability of medical institutions’ websites. In particular, studies on the risk of GLP-1RA misuse or off-label use are scarce and should be investigated.

Regarding future practices, this study suggests that governments should continuously monitor and provide corrective actions on websites, including unscientific content, to promote drug sales. A previous study in Japan showed that the number and volume of newspaper advertisements for dietary supplements that might mislead consumers decreased following the collective action of Japan’s Consumer Affairs Agency [57]. Therefore, government monitoring and regulations are also important to address misleading information provided by medical institutions concerning prescription drug misuse. Furthermore, there are no uniform international guidelines for medical advertising. The permissibility of over-the-counter drug advertisements and advertisements for medical services differs from country to country. However, with the recent proliferation of social networking service platforms, misinformation or information with commercial intent about medicine can spread around the world in a short period of time. Therefore, it is also important to establish international medical advertising guidelines. Second, medical institutions and health care providers should recognize the current problem of GLP-1RA misuse and provide accurate information on the internet and through in-person explanations to individuals who are interested in weight loss. Additionally, medical institutions, academic organizations, and ministries should improve their websites with a focus on search engine optimization to ensure that high-quality medical information appears at the top of search engine results [58]. Internet-based companies, including Google, should be encouraged to prioritize the display of high-quality medical information. Third, medical institutions, health care providers, academic institutions, and ministries should inform citizens that current information on GLP-1RAs on websites is unreliable and that there is no miracle drug that is risk-free and absolutely effective. If individuals without obesity wish to lose weight, they should consult a trusted specialist such as a primary care physician to understand the risks and proceed with the therapy best suited for them.

### Limitations and Strengths

This study has several limitations. First, this study only analyzed Japanese-language websites. The approval status of GLP-1RAs and medical advertising regulations differ from country to country. Thus, the generalizability of the results of this study to other countries is limited. Second, this study assesses the search results for web browsers and websites when viewed with web browsers. The results and website display contents may differ when using a smartphone. The results may have been affected by websites and search algorithms that were optimized for smartphones. Third, this study only used the DISCERN instrument for quality assessment. Therefore, this study was unable to discuss the reliability items other than those evaluated by DISCERN. There are multiple other quality assessment indicators such as the Health on the Net Foundation Code of Conduct certification and the Journal of the American Medical Association (JAMA) benchmark criteria. However, the Health on the Net Foundation Code of Conduct is not well known in Japan, and few websites have obtained it; therefore, it was not used in this study. The JAMA benchmark has only 4 criteria. Since most of the medical institutions of this study were private clinics, it was assumed that the 2 criteria of JAMA benchmark, authorship and attribution, would not differ. Additionally, previous studies that evaluated web-based health information reported a positive correlation between JAMA benchmark scores and DISCERN scores [59,60]. Therefore, only DISCERN was used in this study. Additionally, the DISCERN instrument was developed to enable patients to assess the quality of written information related to treatment choices and to promote the creation of high-quality health information for documents intended for patients. Thus, assessing quality in terms of medical accuracy is not the primary focus of the DISCERN instrument. However, in this study, 2 internal medicine physician coders assessed the medical accuracy when evaluating questions 6, 9, and 12 of the DISCERN instrument, thereby reflecting medical accuracy in the DISCERN score. Fourth, this study relies on a single reviewer to screen eligible websites, which raises the possibility that some potentially eligible websites may have been excluded from the analyzed dataset. Fifth, the intercoder reliability between the 2 coders was ensured to be acceptable; however, coder bias may not have been completely eliminated. Finally, the impact of the websites analyzed in this study on viewers’ cognition and behavior was outside the scope of this study; future studies should examine this aspect.

Despite these limitations, to the best of our knowledge, this is the first study on the quality of information on the websites of medical institutions that prescribe GLP-1RAs for weight loss in Japan. This study provides important implications for future studies and practice on better communication to decrease prescription drug misuse of GLP-1RAs, as discussed above. The quality of the websites of medical institutions that prescribe GLP-1RAs for weight loss was very low, and many of them did not comply with the medical advertising guidelines in Japan. This study shows that many websites, including exaggerated advertisements, may promote the misconception that exercise and dietary therapy are unnecessary. This study also shows that many websites provide biased information that may lead consumers to use GLP-1RAs without presenting alternative weight loss methods other than GLP-1RAs or the option of shared decision-making with health care providers or family members. Consumers and citizens who read such information may misuse GLP-1RAs because of insufficient understanding of their side effect risks and proper usage. Public institutions must monitor and regulate advertising content that violates guidelines and misleads individuals. Health care providers need to inform consumers about the risks and proper usage of GLP-1RAs and provide accurate information related to weight loss. Beneficial medications developed for people’s health should not pose health risks because of the inappropriate information provided by health care providers.

## Appendix

Multimedia Appendix 1 DISCERN instrument.

## Abbreviations

FDA: Food and Drug Administration
GLP-1: glucagon-like peptide-1
GLP-1RA: glucagon-like peptide-1 receptor agonist
JAMA: Journal of the American Medical Association

## Data Availability

The data presented in this study are available upon reasonable request to the corresponding author.
