# Supplementary material for: Quality Assessment of Medical Institutions’ Websites Regarding Prescription Drug Misuse of Glucagon-Like Peptide-1 Receptor Agonists by Off-Label Use for Weight Loss: Website Evaluation Study
Source: JMIR Form Res. 2025 Jan 1;9:e68792. doi: 10.2196/68792 (PMC11736224; doi:10.2196/68792)
Supplement: Multimedia Appendix 1 [file formative_v9i1e68792_app1.doc]

(Appendix 1) DISCERN instrument

SECTION 1. Is the publication reliable?

1. Are the aims clear?
2. Does it achieve its aims
3. Is it relevant?
4. Is it clear what sources of information were used to compile the publication (other than the author or producer?)
5. Is it clear when the information used or reported in the publication was produced?
6. Is it balanced and unbiased?
7. Does it provide details of additional sources of support and information?
8. Does it refer to areas of uncertainty

SECTION 2. How good is the quality of information on treatment choices?

1. Does it describe how each treatment works?
2. Does it describe the benefits of each treatment?
3. Does it describe the risks of each treatment?
4. Does it describe what would happen if no treatment is used?
5. Does it describe how the treatment choices affect overall quality of life?
6. Is it clear that there may be >1 possible treatment choice?
7. Does it provide support for shared decision-making?

SECTION 3. Overall rating of the publication

1. Based on the answers to all of the above questions, rate the overall quality of the publication as a source of information about treatment choices.
